# RETRACTED: Tomassoni et al. Brain Activity of Thioctic Acid Enantiomers: In Vitro and In Vivo Studies in an Animal Model of Cerebrovascular Injury. *Int. J. Mol. Sci.* 2013, *14*, 4580–4595

**DOI:** 10.3390/ijms27104539

**Published:** 2026-05-19

**Authors:** Daniele Tomassoni, Francesco Amenta, Consuelo Amantini, Valerio Farfariello, Lorenzo Di Cesare Mannelli, Innocent E. Nwankwo, Carlotta Marini, Seyed Khosrow Tayebati

**Affiliations:** 1 School of Biosciences and Biotechnologies, University of Camerino, 62032 Camerino, Italy; daniele.tomassoni@unicam.it; 2 School of Medicinal Sciences and Health Products, University of Camerino, 62032 Camerino, Italy; francesco.amenta@unicam.it (F.A.); consuelo.amantini@unicam.it (C.A.); valerio.farfariello@uniroma1.it (V.F.); nwankwoejyki@yahoo.com (I.E.N.); 3 Department of Urology and Andrology, University of Perugia, 06129 Perugia, Italy; 4 Department of Preclinical and Clinical Pharmacology, University of Florence, 50139 Florence, Italy; lorenzo.mannelli@unifi.it; 5 School of Veterinary Medical Sciences, University of Camerino, 62024 Matelica, Italy

The journal retracts the article, “Brain Activity of Thioctic Acid Enantiomers: *In Vitro* and *in Vivo* Studies in an Animal Model of Cerebrovascular Injury” [1].

Following publication, concerns were brought to the attention of the Editorial Office regarding the integrity of a number of images presented in this publication [1].

Adhering to our standard procedure, an investigation was conducted by the Editorial Office and Editorial Board that identified indications of inappropriate editing and partial duplication between Figures 4 and 5 of this publication [1]. While the authors acknowledged these concerns and fully collaborated within this process, the explanations and supporting materials provided were not deemed sufficient by the Editorial Board to resolve the identified concerns. As a result, the Editorial Board has lost confidence in the reliability of the findings, and has decided to retract this publication, as per MDPI’s retraction policy.

This retraction was approved by the Editor-in-Chief of the journal *IJMS*.

The authors have been informed of this retraction and have indicated their disagreement with this decision.
